# Perceptions of the motivational climate, basic psychological needs, and life skills development in Chinese physical education students

**DOI:** 10.3389/fpsyg.2023.1232849

**Published:** 2023-08-15

**Authors:** Shaofeng Zheng, Xiangbo Ji, Liping Cheng, Jianhua Xu, Lorcan Donal Cronin

**Affiliations:** ^1^Department of Physical Education, The Open University of Fujian, Fuzhou, China; ^2^School of Physical Education and Sport Science, Nanjing Normal University, Nanjing, China; ^3^School of Physical Education and Sport Science, Fujian Normal University, Fuzhou, China; ^4^Mary Immaculate College, Limerick, Ireland

**Keywords:** motivational climate, achievement goal theory, self-determination theory, basic psychological needs, life skills, physical education

## Abstract

**Introduction:**

Life skills can have a positive impact on young people’s mental health, academic performance, and overall well-being. Physical education (PE) is viewed as a promising setting for developing students’ life skills, but less is known about this in non-English speaking countries such as China. Based on the integration of Self-Determination Theory (SDT) and Achievement Goal Theory (AGT), we aimed to examine the relationships between students’ perceptions of the teacher-initiated motivational climate (mastery- or performance-oriented) and their life skills development in PE, as well as the mediating role of their basic psychological needs (BPNs) (satisfaction or frustration).

**Methods:**

We employed a cross-sectional survey. Chinese students (*N* = 533, Age range = 13–18 years) completed measures assessing these variables. We fulfilled correlational and mediational analyses.

**Results:**

These findings showed that mastery climate was positively associated with needs satisfaction (*r* = 0.66) and eight life skills (*r* range = 0.44–0.61), whereas negatively associated with needs frustration (*r* = −0.49). Performance climate was positively related to needs frustration (*r* = 0.52), but negatively related to needs satisfaction (*r* = −0.38) and eight life skills (*r* range = −0.28 – −0.15). Needs satisfaction was positively (*r* range = 0.44–0.65), while needs frustration was negatively (*r* range = −0.50 – −0.34) linked with eight life skills. Furthermore, needs satisfaction positively mediated the effect of mastery climate on life skills development, but it is not found that needs frustration mediated the effect of performance climate on life skills besides goal setting, social skills, and time management.

**Conclusion:**

In conclusion, our study extended the previous literature on life skills in PE, and highlighted the roles of motivational climate and BPNs on students’ life skills development. In practice, PE teachers should be encouraged to create a mastery climate as well as avoid a performance climate, to foster students’ BPNs satisfaction, which in turn, promote their life skills development.

## Introduction

One key aspect of Positive Youth Development (PYD) is the area of life skills development through sports and physical education (PE) (Goudas and Giannoudis, 2008; Mossman et al., 2021). Life skills are described as functional skills that individuals develop and use effectively in one context (such as sports, physical education, home, and community) and that are then used effectively in other contexts (Williams et al., 2020). Others have further suggested that life skills are adaptive and positive behaviors that empower individuals to deal effectively with the demands and challenges of everyday life (Singla et al., 2020). Furthermore, life skills are important predictors of young people’s future well-being, academic performance, and job satisfaction (Zins et al., 2004; De Ridder et al., 2018).

In English-speaking Western countries, research on life skills development has been extensive. In contrast, there has been little research on life skills development beyond such countries (Santos et al., 2016; Opstoel et al., 2019). This is an important point as differences between countries and cultures may affect student’s learning of life skills. For example, higher mean scores for life skills development have been seen for Chinese (Ji et al., 2022) as compared to British (Cronin et al., 2018) PE students (3.66 versus 3.18 on the 1–5 response scale of the Life Skills Scale for PE; Cronin et al., 2018). As such, it is essential to study how teachers’ behaviors and practices impact students’ life skills development through PE in a variety of countries and cultures (Ji et al., 2022). To begin to address Opstoel et al.’s (2019) proposition that life skills development in PE research should be extended to other continents, countries, and cultures, our study addresses how Chinese students develop their life skills in PE.

### The development of life skills in PE

Extracurricular activities that can prompt young people to develop their life skills include PE, sports, music, and drama (Holt et al., 2017; Opstoel et al., 2019). Of the extracurricular activities that have been studied, sports and PE have some of the greatest effects on the development of young people’s life skills (Marsh, 1992; Larson et al., 2006; Cronin and Allen, 2017). The majority of this research has focused particularly on youth sports, which has proven to be an excellent avenue for enhancing youth development (Gould and Carson, 2008; Cronin and Allen, 2017; Holt et al., 2017; Williams et al., 2020). Less research on the development of life skills has concentrated on PE. Nevertheless, some past research has shown PE to be an effective setting for promoting students’ life skills development (e.g., Hodge et al., 2013; Pesce et al., 2016; Cronin et al., 2020). For example, life skills programs integrated with a multi-sports PE setting have positive effects on cognitive outcomes such as executive function, tactical cooperation skills, and decision-making (Pesce et al., 2016). Goudas et al. (2006) argued that PE is a widespread activity in our society, with which most students are acquainted; as such, life skills can be learned along with physical skills through demonstration and practice in PE. In line with such a proposition, educational policies and past research have indicated that life skills can and should be part of the PE curriculum (Jacobs et al., 2022).

Within China, the purpose of an ongoing educational revolution is the promotion of students’ overall development and health (The Ministry of Education of the People’s Republic of China, 2018). Under this educational reform (The Ministry of Education of the People’s Republic of China, 2018), the PE curriculum has been revised to include life skills development as one of the key aims of PE classes. Because of this, there is a pressing need to explore students’ perceptions and experiences about learning life skills within the Chinese PE context.

### How to develop life skills

Researchers in sport psychology, PE and PYD have particularly focused on how life skills can be taught and developed through sports and PE (Pierce et al., 2017). Two major approaches to life skills development have been identified as the implicit and the explicit approach (Turnnidge et al., 2014). Specifically, an implicit approach involves the conditions that coaches or teachers put in place to facilitate life skills development and transfer, without those delivering the program having to discuss these explicitly; an example would be a well-structured sports environment that facilitates a PYD climate, fosters positive relationships, and models positive behaviors (Turnnidge et al., 2014; Chinkov and Holt, 2016; Bean et al., 2018). In comparison, an explicit approach involves delivering a life skills program by drawing upon specific pedagogical strategies; these could include providing feedback, discussing life skills applications, helping young people make an action plan, and creating opportunities to practise life skills (Turnnidge et al., 2014; Bean et al., 2018).

Although the explicit approach can be effective (Williams et al., 2020), Pierce et al. (2017) highlighted the importance of an implicit approach. They argued that the absence of deliberate strategies for teaching life skills does not necessarily equate to the absence of life skills development for participants. For example, the social interactions fostered during participation in team sports were conducive to learning life skills, despite the coaches not teaching life skills directly (Holt et al., 2008). Furthermore, the implicit approach toward life skills development is less time-consuming and more manageable than adopting an explicit approach (Bean and Forneris, 2016; Chinkov and Holt, 2016). Indeed, young people are likely to acquire life skills through an implicit approach from participation in sports or PE, even if the teaching of such skills is not intentional or the goal of sports activities (Malete et al., 2022). As PE and sports environments share many similarities (Smith et al., 2016), research from sports also helps to explain how to develop students’ life skills in PE.

### Self-determination theory and life skills development

There are several theories available for investigating the key psychological mechanisms that affect life skills development. The most frequently discussed theory regarding life skills development is Self-Determination Theory (SDT; Ryan and Deci, 2017). SDT is best described as a theory of motivation, health, well-being, and optimal development (Deci and Ryan, 2008; Ryan and Deci, 2008). A key component of SDT is the degree to which the three basic psychological needs (BPNs) of autonomy, competence, and relatedness are satisfied or frustrated (Haerens et al., 2015). Autonomy satisfaction involves the student feeling empowered and self-directed in their behavior; competence satisfaction refers to the student feeling effective in the PE environment; and relatedness satisfaction involves the student having warm and caring relationships with fellow students and the teachers (Cheon et al., 2016). Conversely, autonomy frustration pertains to the student feeling pressured or forced to take part in activities, competence frustration involves feeling ineffective or inadequate in PE, and relatedness frustration refers to the student feeling rejected or excluded by fellow students or teachers (Cheon et al., 2016). Ryan and Deci (2008) contend that when these three BPNs are satisfied, people experience positive psychological development. In terms of life skills development, Hodge et al. (2013) proposed a life skills intervention model which argued that the more the three BPNs are satisfied and internalized by the participant, the more likely they will develop their life skills.

Another key component of SDT is the social-environmental factors (Ryan and Deci, 2017) that are created by significant others (e.g., teachers, coaches, peers, parents) and which are viewed as essential for satisfying the three BPNs (Williams et al., 1996; Baard et al., 2004; Moreau and Mageau, 2012). Importantly, a positive environmental climate is said to predict the internalization (i.e., development) and generalization (i.e., transfer) of life skills (Hodge et al., 2013; Pierce et al., 2017). Teachers or coaches who create a positive social environment play a pivotal role in implicitly facilitating the development of both intrapersonal (e.g., self-control and effort) and interpersonal (e.g., teamwork and social responsibility) life skills (Sheridan et al., 2014; Newman et al., 2020; Santos et al., 2020). Gould and Carson (2008) defined teachers and coaches as important external assets through which individual participants enter sports or PE. These external assets can facilitate or hinder the learning of life skills and must be recognized as important socializing agents on participants, either through their direct or indirect influences (Pierce et al., 2017). In contrast, Kendellen and Camire (2015) indicated that teachers’ inappropriate social interactions (e.g., favoritism, superiority) may thwart the ability of youth to learn life skills.

Hodge et al. (2013, 2016) incorporated the key tenets of SDT in their conceptual model of life skills development, suggesting that a positive social-environmental climate contributes to satisfying participants’ three BPNs, and, in turn, helps them to acquire life skills. Cronin et al. (2018, 2019, 2020) have subsequently used this SDT-based model as a theoretical framework for researching how to develop students’ life skills in PE. Their first study (Cronin et al., 2018) highlighted that an autonomy-supportive teaching climate positively predicted PE students’ life skills development and psychological well-being. In their second study, Cronin et al. (2019) found that an autonomy-supportive teaching climate was positively related to students’ development of eight life skills, through meeting students’ BPNs. Finally, using a two-wave longitudinal design, their third study (Cronin et al., 2020) suggested that satisfaction of students’ needs (measured in the middle of the school term) positively predicted their development of the same eight life skills at the end of the school term. According to the latter two studies (Cronin et al., 2019, 2020), satisfaction with whether their total psychological needs (the sum of the three BPNs) are met has more influence on participants’ life skills development than each of the three BPNs in isolation. This has also been highlighted by Sheldon and Niemiec (2006) and Deci and Ryan (2014), who proposed that the sum of the three BPNs, or the balance between them, was vital for promoting psychological development. In summary, the interpretation of the above-mentioned results supported the effectiveness of SDT for investigating students’ learning of life skills in PE.

### The motivational climate and life skills development

Previous research has demonstrated that a needs-supportive environment (e.g., autonomy-supportive teaching climate) can satisfy students’ BPNs, and, in turn, facilitate their learning of life skills in PE (e.g., Cronin et al., 2019). However, the motivational climate within sports also predicts the development of life skills (Petitpas et al., 2005; Sackett and Gano-Overway, 2017). The motivational climate is created and fostered by significant others, such as PE teachers or (in achievement environments) the coach (Chu and Zhang, 2018; Chen et al., 2020). It consists of two opposing types of climates: a mastery-oriented climate and a performance-oriented climate. A mastery-oriented climate is one in which the standards of success and failure are measured in relation to oneself and the process of improvement is valued over the achievement of specified results. In contrast, a performance-oriented climate is one in which the standards of success and failure lie with others and a sense of competence is gained through comparison with others (Ames, 1992; Maro and Roberts, 2012). The motivational climate is included in Achievement Goal Theory (AGT; Ames, 1992), which has dominated research on achievement motivation in sports and physical activity over the past 30 years (Harwood et al., 2015). This research has suggested that a mastery-oriented climate – which facilitates participants’ motivation, focuses on individuals’ efforts and improvements, and promotes cooperation among peers – is more likely to lead to adaptive outcomes (e.g., Treasure and Roberts, 2001; Maro and Roberts, 2012; Brown and Fry, 2014). In contrast, a performance climate tends to lead to participants’ attention being focused on beating others and showing their ability (Roberts et al., 2012), which is more likely to produce maladaptive outcomes such as antisocial behaviors (Hodge and Gucciardi, 2015), negative affect (Gould et al., 2012), anxiety, and reduced enjoyment for participants (Gjesdal et al., 2019). Some previous findings have also revealed that a mastery-oriented climate has a positive effect on life skills development in youth sports, while a performance climate harms the development of some life skills (Gould et al., 2012; Mossman et al., 2021). However, no previous research has investigated how the two types of motivational climates created by teachers affect students’ life skills development in the PE context.

### The integration model of SDT and AGT in PE

Several PE research has demonstrated teachers’ autonomy- or need-supportive teaching behavior can positively predict students’ BPNs satisfaction (e.g., Cronin et al., 2019; Leo et al., 2020), and then foster positive outcomes such as autonomous motivation and life skills development. In contrast, teachers’ controlling or need-thwarting teaching behavior negatively predict their BPNs frustration (e.g., Cronin et al., 2019; Leo et al., 2020), and lead to negative influences. Within a mastery climate, teachers like to provide different tasks for students to choose, recognize their effort and self-improvement, focus on each student performance and get them all feel play important role in PE. These behaviors make them feel more safe, confident, valuable and within self-control, which would foster student perceive more autonomy, competence, and relatedness supporting (Duda, 2001; Reinboth and Duda, 2006). Within a performance climate, teachers like to compare and emphasize on transcending others, punish their errors, only appreciate outstanding performers and good grade. These behaviors make them feel more pressure, inferior, competitive, and out of self-control, which would make them perceive more autonomy, competence, and relatedness thwarting (Duda, 2001; Reinboth and Duda, 2006). Consequently, we can speculate that mastery climate would be a positive predictor of BPNs satisfaction, while performance climate be a positive predictor of BPNs frustration. Given certain similarities between SDT and AGT (Chen et al., 2020; Girard et al., 2021), which both take account of the interplay between contextual and individual variables, some studies have called for the integration of the two models, to investigate the relationships between the motivational climates proposed by AGT, students’ BPNs based on SDT, and cognitive or behavioral outcomes in PE (Standage et al., 2003; Jaakkola et al., 2015).

Previous research has shown that a mastery climate, as created by PE teachers, was positively associated with adaptive outcomes such as higher PE grades (Rodrigues et al., 2020), greater intention to engage in physical activity (Di Battista et al., 2019), higher levels of moderate to vigorous physical activity (MVPA) (Chen et al., 2020), and improved concentration (Mastagli et al., 2021). Furthermore, psychological needs mediated the relationship between a mastery climate and these adaptive outcomes. In contrast, a performance climate was associated with a reduced experience of needs satisfaction and other negative consequences such as lower PE grades (Rodrigues et al., 2020) and goal-related frustrations and boredom (Serrano et al., 2016; Garca-Gonzlez et al., 2019). That is, a tendency to be mastery-oriented would positively predict BPNs and positive outcomes, while being performance-oriented would have the opposite effect (Rodrigues et al., 2020). Nonetheless, scant research has explained the relationships between the two motivational climates, psychological needs frustration, and cognitive or behavioral outcomes in PE. Moreover, to the best of our knowledge, no studies in PE have investigated the differential roles of the two motivational climates on BPNs and students’ life skills development. Based on the above studies, it was hypothesized that, within PE, a mastery-oriented climate would have positive effects on students’ life skills development, whereas a performance climate would be more likely to hinder it.

### The present study

**Figure 1:**
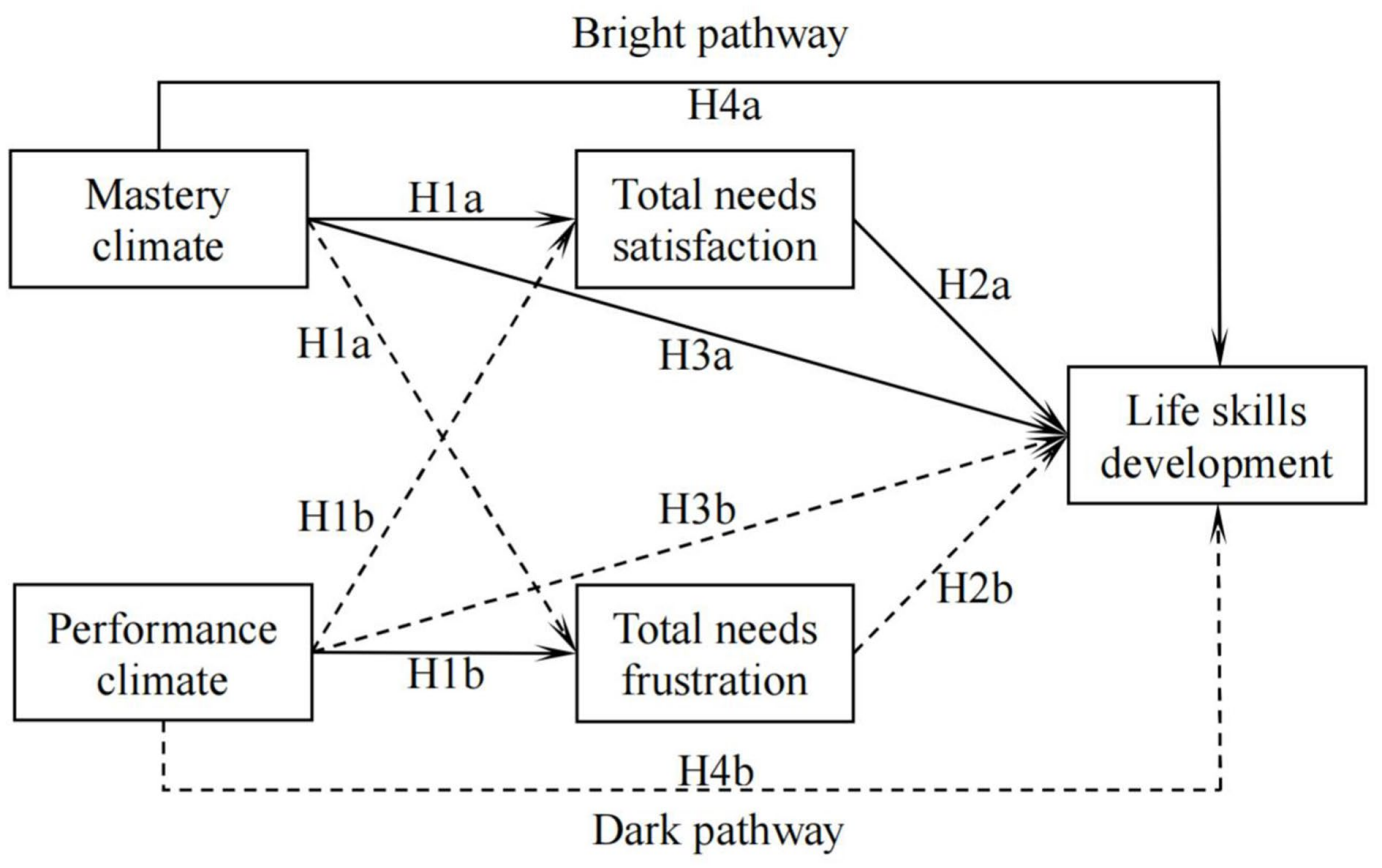
The hypothesized model, including mastery and performance climates, needs satisfaction and frustration, and life skills development. Continuous arrows show positive relationships, while discontinuous arrows show negative relationships. H1a, Hypothesis 1a; H1b, Hypothesis 1b; etc.

Using SDT and AGT as our theoretical framework (Standage et al., 2003; Jaakkola et al., 2015), the purpose of this study was to examine the relationship among the teacher-initiated motivational climates, students’ satisfaction versus frustration with their BPNs, and their life skills development in PE. We assessed both the bright pathways of SDT (Hodge et al., 2013; Cronin et al., 2019; Rodrigues et al., 2020) as well as the dark pathways (De Meyer et al., 2016; Liu et al., 2017), while also investigating the specific cross-paths of SDT (Vansteenkiste and Ryan, 2013). According to the previous research, we proposed four hypotheses for the study (see Figure 1).

First, we expected that students’ perceptions of a mastery climate would be positively associated with total needs satisfaction and negatively with total needs frustration (Hypothesis 1a), while a performance climate would correlate positively with total needs frustration and negatively with total needs satisfaction (Hypothesis 1b). Second, we hypothesized that life skills development would be positively associated with total needs satisfaction (Hypothesis 2a), but negatively correlated with total needs frustration (Hypothesis 2b). Third, we hypothesized that a mastery climate would be positively related to life skills development (Hypothesis 3a), whereas a performance climate would be negatively related to life skills development (Hypothesis 3b). Finally, we hypothesized that total needs satisfaction would mediate the positive relationship between students’ perceptions of a mastery climate and life skills development in PE (Hypothesis 4a), whereas total needs frustration would mediate the negative relationship between a performance climate and students’ life skills development (Hypothesis 4b).

## Methods

### Participants

A total of 559 students from five regions of China participated in this study. These students completed a survey containing measures assessing the motivational climate, satisfaction and frustration with their BPNs, and their life skills development in PE. However, 26 students were excluded from the final sample because they responded several times to the same item, failed to answer more than five items in a row or failed to answer more than a fourth of the items. The final sample included 533 PE students (259 males and 274 females) between 13 and 18 years of age (*M*age = 15.36; *SD* = 1.28). The students participated in PE for an average of 111 min per week (*SD* = 24.50) and were taking PE as an exam subject. In total, 19 classes from 8 middle and high schools were included; on average, there were 28 students per class. In their PE classes, the students took part in a variety of sports, including badminton, basketball, cheerleading, football, gymnastics, martial arts, rope skipping, table tennis, track and field, tai chi, and volleyball. Furthermore, 64.1% of students participated in extracurricular sports outside of PE, which included students participating in 1–3 different sports for an average of 92.41 min per week (*SD* = 82.34; range = 30–420).

### Procedures

When recruiting students for our study, the first author contacted administrators and PE teachers from the students’ schools and explained the purpose of the study, so that approval for the study could be obtained. Each student that participated also provided written informed consent before completing the survey. To ensure their responses were as independent, accurate, and honest as possible, participants were provided with standardized written and verbal instructions and were informed that there were no right or wrong answers and that their responses were anonymous and confidential. The inclusion criteria for the study were that participants were middle and high school students who regularly participate in PE classes. In China, students who take part in PE are usually 13–15 years old in middle school, and 16–18 in high school students. The data collection was conducted in their PE classes or self-study classes after the middle of the spring term, to ensure that students had sufficient experience with the variables that would be addressed in the study. Most participants took 15–20 min to complete the survey.

### Measures

#### Perceived motivational climate

The students’ perceptions of the teacher-initiated motivational climate during their PE lessons were measured via a modified Chinese version of the Motivational Climate Scale for Youth Sports (MCSYS) (Smith et al., 2008; Wu et al., 2021). In line with Weeldenburg et al. (2020), the wording of the scale was slightly adapted to the PE context. It consists of six items measuring perceptions of a mastery climate (e.g., ‘The PE teacher told us to help each other improve skills’) and six items measuring perceptions of a performance climate (e.g., ‘The PE teacher will punish us if we make a mistake’). Students responded to items on a 5-point scale ranging from 1 (‘not at all true for me’) to 5 (‘very true for me’). Total scores for each scale were computed by averaging the individual item scores. Wu et al. (2021) revised this scale for Chinese adolescents and provided evidence for its internal consistency and test–retest reliability. In the current study, the Cronbach’s alpha coefficients for the mastery and performance-oriented dimensions were 0.93 and 0.80, respectively.

#### Basic needs satisfaction and frustration

The Chinese version of the psychological needs satisfaction scale for PE (PNSSPE) (Liu and Chung, 2014) and the psychological needs frustration scale for PE (PNTSPE) (Liu and Chung, 2015) were used to assess Chinese students’ needs satisfaction and frustration. The item stem for the two scales was “In my physical education classes, I..”. The PNSSPE includes 10 items which measure: autonomy satisfaction (e.g., “free to do physical activities the way I like”), competence satisfaction (e.g., “have the ability to perform well”), and relatedness satisfaction (e.g., “get along well with the people”). The PNTSPE includes nine items which measure: autonomy frustration (e.g., “often feel like I have to follow other people’s commands”), competence frustration (i.e., “often feel like I’m inadequate”) and relatedness frustration (e.g., “feel some people do not like me much”). Participants responded to the items using a 7-point scale (1 = strongly disagree, 7 = strongly agree). The two scales have been shown by previous studies to have good internal consistency and reliability among Chinese students (Liu and Chung, 2014; Liu and Chung, 2015; Liu et al., 2017). Sheldon and Niemiec (2006), along with Deci and Ryan (2014), have highlighted that participants’ sum or balance of the three BPNs is vital for promoting positive psychological development. Camire et al. (2019) have also called for studies to examine the relationships between satisfaction of total BPNs and young people’s life skills development, and other studies have highlighted the importance of this relationship as well(Hodge et al., 2013; Cronin et al., 2020). As such, the current study combined the three BPNs to create a total measure of needs satisfaction and frustration scores. In this study, the Cronbach’s alpha coefficient for the total needs satisfaction subscales was 0.92, and 0.93 for the total needs frustration subscales.

#### Life skills development

The 43-item Chinese version of the Life Skills Scale for PE (LSSPE; Ji et al., 2022) was used to measure how students perceived their own life skills development. The item stem for this scale was: “PE classes have taught me to..”. Example items included: teamwork (7 items; e.g., “help build team/group spirit); goal setting (7 items; e.g., “set specific goals”); social skills (5 items; e.g., “maintain close friendships”); problem-solving and decision-making (4 items; e.g., “evaluate a solution to a problem”); emotional skills (4 items; e.g., “Notice how I feel”); leadership (8 items; e.g., “know how to motivate others”); time management (4 items; e.g., “control how I use my time”) and interpersonal communication (4 items; e.g., “Pay attention to what someone is saying”). Students responded to items on a 5-point scale ranging from 1 (“not at all”) to 5 (“very much”). The subscales also demonstrated strong internal consistency and reliability in previous studies with Chinese students (Ji et al., 2022). In this study, the Cronbach’s alpha coefficients for the eight life skills subscales ranged from 0.81 to 0.96.

### Data analysis

For our preliminary analyses, missing values, skewness and kurtosis, descriptive statistics and correlations between the study variables were calculated using SPSS Version 25.0 (IBM Corporation, 2017). To decide on whether to conduct mediation analysis, we first assessed whether significant correlations existed between our independent, mediator, and dependent variables. Given that individual differences may affect life skills development (Gould and Carson, 2008), we assessed potential gender and age group (13–15 versus 16–18 years old) differences on all variables, to decide whether to control for these variables in the mediation analyses.

For the mediation analyses, we used the PROCESS macro for SPSS (Hayes, 2013). Specifically, we used model number four of the macro and we included 20,000 bootstrap resamples and a 95% bias-corrected confidence interval (CI), which can be appropriate for smaller sample sizes (Preacher and Hayes, 2004). In addition, Our sample size surpassed the median sample size for cross-sectional studies in sport and exercise psychology (Schweizer and Furley, 2016) and for mediation analyses in major psychology journals (Sim et al., 2021). Given the difference on indirect effect and mediation (Hayes, 2013), we assess the indirect effect of each mediator after judging whether mediation was evident. When the mediators are included in the model, mediation occurs when a statistically significant regression coefficient (*p* < 0.05) for the total effect reduces in value for the direct effect (Hayes, 2013). Specifically, when this reduction results in a non-significant regression coefficient for the direct effect, full mediation is said to be occurring. In comparison, when the regression coefficient for the direct effect is reduced but remains significant, partial mediation is said to be occurring. An indirect effect is evident when zero is not included within the lower and upper bound Cis for a possible mediator (Hayes, 2013).

With regard to effect sizes, according to Cohen’s (1988) criteria, we judged the correlations as either small (*r* = ± 0.10 to ±0.29), medium (*r* = ± 0.30 to ±0.49), or large (*r* > ± 0.50). *R*^2^ values for the mediation model were converted to Cohen’s *f*^2^ (a measure of effect size) using this formula (*R*^2^/(*1–R*^2^)) and can likewise be identified as small (*f*^2^ ≥ 0.02), medium (*f*^2^ ≥ 0.15), or large (*f*^2^ ≥ 0.35) (Cohen, 1988).

## Results

### Preliminary analysis

Missing value analysis found that the percentage of missing data in the sample was very low (0.3%). As a result, we performed a mean substitution to minimize lost cases. We also calculated skewness and kurtosis values as a means of assessing the normality of the main study variables. The skewness values ranged from −0.97 to 0.50, while the kurtosis values ranged from −0.57 to 0.48 (See Table 1), indicating that the data approximated a normal distribution (Tabachnick and Fidell, 2013).

**Table 1:** Mean scores, standard deviations, reliability coefficients, skewness values, kurtosis values and correlations for all study variables.

	1	2	3	4	5	6	7	8	9	10	11	12
(1)Mastery climate	–											
(2)Performance climate	−0.34**	–										
(3)Total needs satisfaction	0.66**	−0.38**	–									
(4)Total needs frustration	−0.49**	0.52**	−0.61**	–								
(5)Teamwork	0.56**	−0.28**	0.59**	−0.43**	–							
(6)Goal setting	0.53**	−0.21**	0.55**	−0.42**	0.69**	–						
(7)Social skills	0.48**	−0.24**	0.55**	−0.45**	0.58**	0.54**	–					
(8)Problem solving	0.50**	−0.25**	0.50**	−0.40**	0.64**	0.66**	0.61**	–				
(9)Emotional skills	0.45**	−0.21**	0.44**	−0.36**	0.47**	0.52**	0.49**	0.58**	–			
(10)Leadership	0.50**	−0.21**	0.57**	−0.40**	0.68**	0.65**	0.65**	0.70**	0.57**	–		
(11)Time management	0.44**	−0.15**	0.45**	−0.36**	0.59**	0.62**	0.52**	0.66**	0.54**	0.65**	–	
(12)Communication	0.44**	−0.27**	0.44**	−0.34**	0.52**	0.50**	0.62**	0.57**	0.54**	0.63**	0.53**	–
Scale range	1–5	1–5	1–7	1–7	1–5	1–5	1–5	1–5	1–5	1–5	1–5	1–5
Mean score	4.35	2.09	5.32	2.67	3.49	3.54	3.65	3.68	3.72	3.50	3.39	3.887
Standard deviation	0.69	0.76	1.06	1.15	0.78	0.92	0.87	0.83	0.72	0.79	0.91	0.778
Cronbach’s alpha	0.93	0.80	0.92	0.93	0.92	0.96	0.90	0.92	0.81	0.91	0.90	0.877
Skewness value	−0.974	0.5	−0.308	0.317	0.1	−0.372	−0.202	−0.257	−0.103	0.034	−0.053	−0.222
Kurtosis value	0.476	−0.077	−0.144	−0.565	−0.163	0.087	−0.431	−0.095	0.01	−0.193	−0.169	−0.485

*N* = 533. **p < 0.01. Problem solving, problem-solving and decision making; Communication, interpersonal communication skills.

For assessing potential gender and age group differences on all variables, results showed that there were age group differences (*p* < 0.01) for the study variables for all eight life skills and total needs satisfaction, while there were gender differences (*p* < 0.01) for the performance climate and time management skills. Therefore, we controlled for age group and gender in our mediation analyses.

### Descriptive statistics

Table 1 displays the scale ranges, mean scores, standard deviations, reliability coefficients, skewness values, kurtosis values and correlations for the study variables. The Cronbach’s alpha values for all variables were above 0.80, which indicates adequate internal reliability. The correlations between the study variables were consistent with our expectations. On the bright pathway, the mastery climate was positively and significantly correlated with participants’ total needs satisfaction (*r* = 0.66, *p* < 0.01) and the development of all eight life skills (*r* range = 0.44–0.61, all *p* < 0.01). Total needs satisfaction was positively and significantly related to students’ development of all eight life skills (*r* range = 0.44–0.65, all *p* < 0.01). On the dark pathway, the performance climate was positively and significantly related to total needs frustration (*r* = 0.52, *p* < 0.01), while it was negatively and significantly correlated with students’ development of all eight life skills (*r* range = −0.28 – −0.15, all *p* < 0.01). Total needs frustration was negatively and significantly associated with students’ development of all eight life skills (*r* range = −0.50 – -0.34, all *p* < 0.01). Regarding the cross paths, the mastery climate was negatively and significantly correlated with participants’ total needs frustration (*r* = −0.49, *p* < 0.01), whereas the performance climate was negatively and significantly correlated with total needs satisfaction (*r* = −0.38, *p* < 0.01). Furthermore, mastery climate had lager effect size on correlations with all eight life skills than performance climate. The results of the correlational analysis meant that we conducted further the mediation analyses for both the bright and the dark pathways.

### Mediation analysis

**Figure 2:**
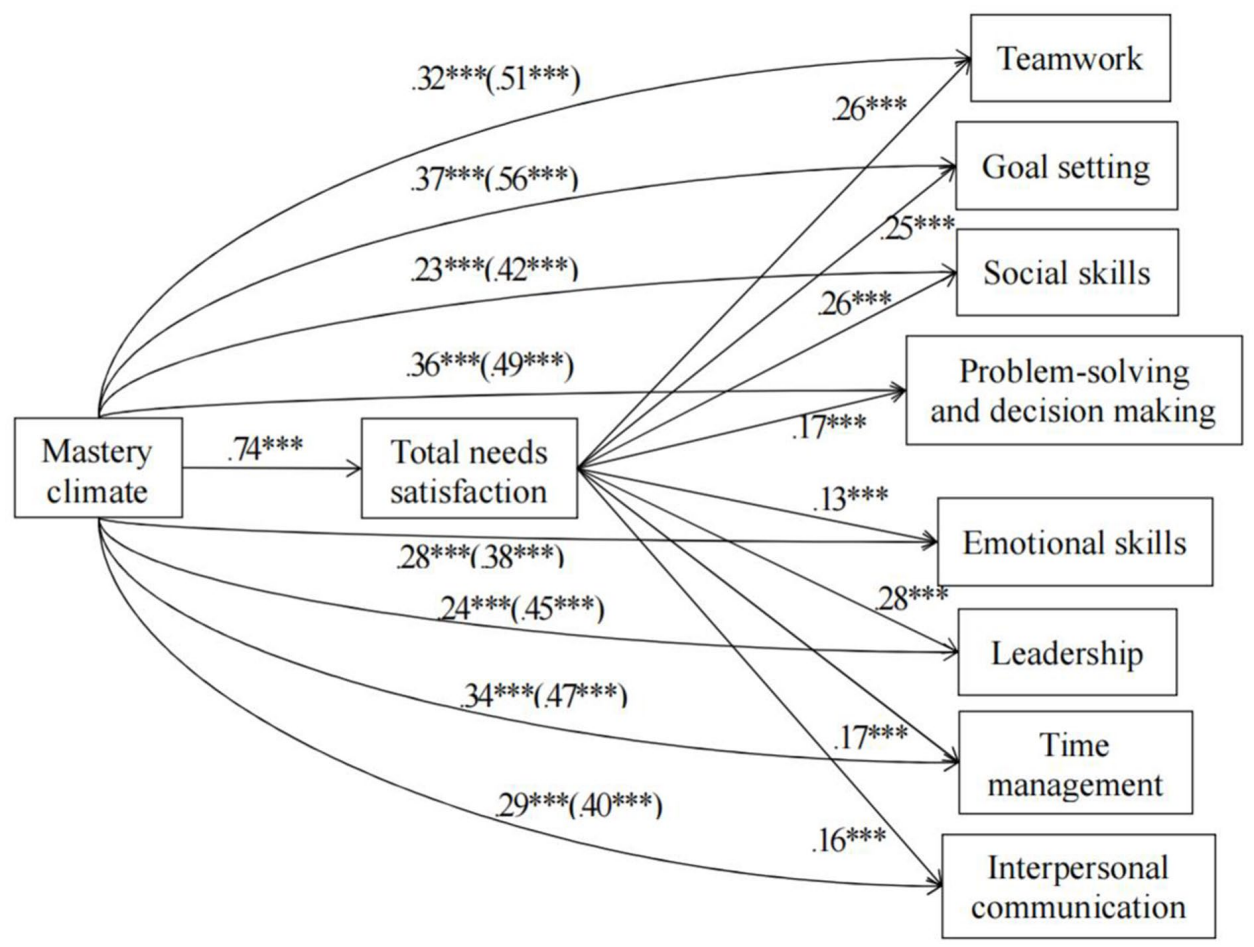
Model including mastery climate and the eight life skills. Values signify unstandardized regression coefficients. The direct effect of mastery climate on each of the life skills is outside the parentheses whereas the total effect is inside the parentheses. Performance climate, total needs frustration, gender, and age group were entered as covariates in all models. ***p < 0.001.

**Figure 3:**
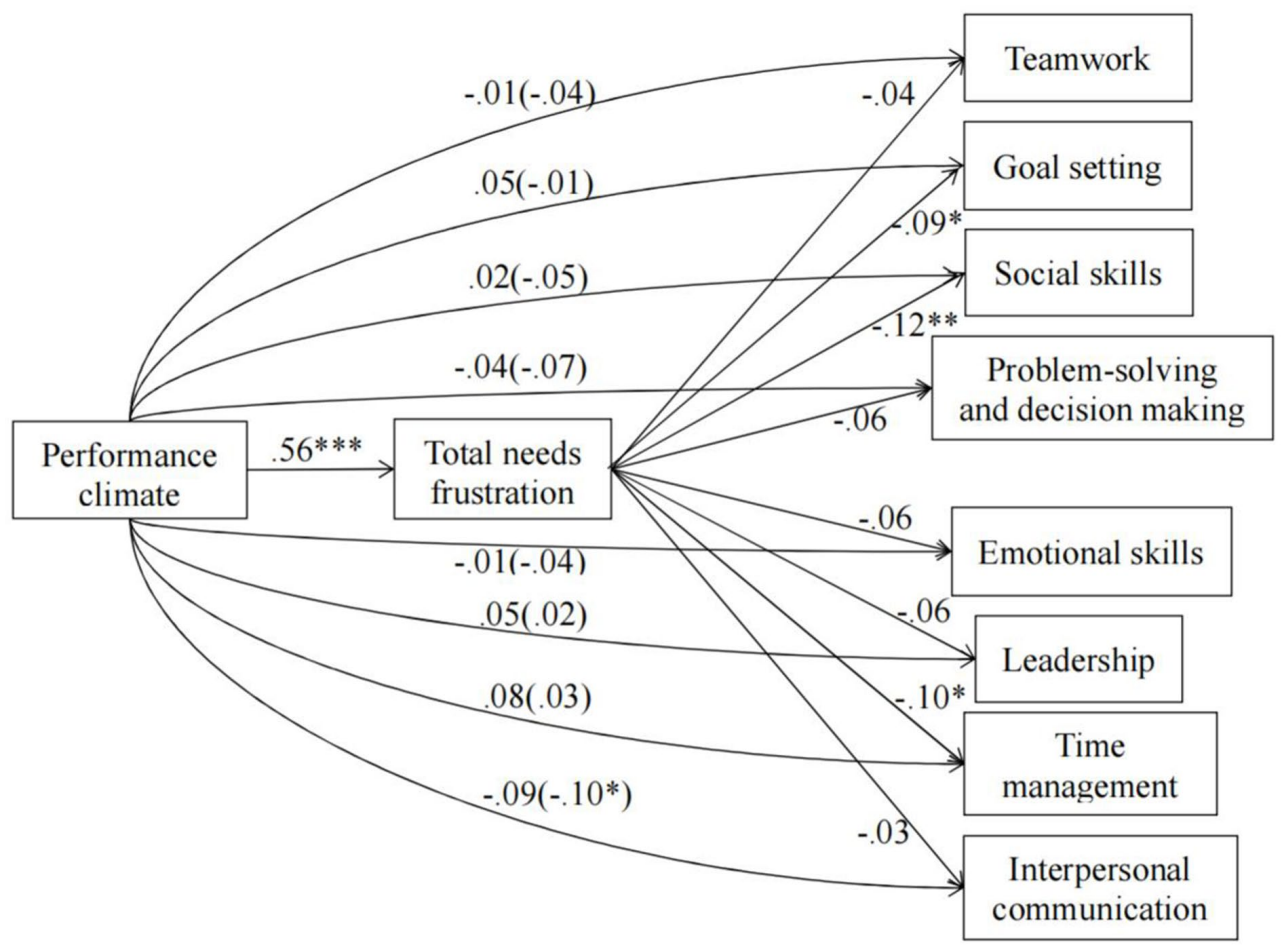
Model including performance climate and the eight life skills. Values signify unstandardized regression coefficients. The direct effect of performance climate on each of the life skills is outside the parentheses whereas the total effect is inside the parentheses. Mastery climate, total needs satisfaction, age group, and gender were entered as covariates in all models. *p < 0.05, **p < 0.01, ***p < 0.001.

Figures 2, 3 display the mediation models with unstandardized regression coefficients that we tested. In these mediation models, we included the mastery and performance climates as the independent variables, total needs satisfaction and frustration as the mediators, and the eight life skills as the dependent variables. Tables 2, 3 shows the indirect effect of mastery climate or performance on students’ each life skills development through total needs satisfaction or frustration.

**Table 2:** Indirect effect of mastery climate on students’ each life skills development through total needs satisfaction.

	Bootstrap effect	Bootstrap SE	95% CI
Teamwork	0.19	0.04	[0.12, 0.27]
Goal setting	0.19	0.05	[0.1, 0.29]
Social skills	0.20	0.04	[0.13, 0.28]
Problem solving	0.12	0.04	[0.06, 0.19]
Emotional skills	0.09	0.03	[0.03, 0.16]
Leadership	0.21	0.03	[0.15, 0.28]
Time management	0.13	0.04	[0.06, 0.21]
Communication	0.12	0.03	[0.06, 0.18]

*N* = 533. Age group, gender, performance climate, total needs frustration were entered as covariates in all models. Twenty thousand bootstrap resamples and 95% bias corrected confidence intervals were employed. Cl, confidence interval; Problem solving, problem-solving and decision making; Communication, interpersonal communication skills.

**Table 3:** Indirect effect of performance climate on students’ each life skills development through total needs frustration.

	Bootstrap effect	Bootstrap SE	95% CI
Teamwork	−0.02	0.02	[−0.06, 0.01]
Goal setting	−0.05	0.02	[−0.1, −0.01]
Social skills	−0.07	0.02	[−0.11, −0.03]
Problem solving	−0.03	0.02	[−0.08, 0.01]
Emotional skills	−0.04	0.02	[−0.08, 0.01]
Leadership	−0.03	0.02	[−0.07, 0.01]
Time management	−0.05	0.02	[−0.1, −0.01]
Communication	−0.02	0.02	[−0.05, 0.02]

*N* = 533. Age group, gender, mastery climate, total needs satisfaction were entered as covariates in all models. Twenty thousand bootstrap resamples and 95% bias corrected confidence intervals were employed. Cl, confidence interval; Problem solving, problem-solving and decision making; Communication, interpersonal communication skills.

For the models in Figure 2, the mastery climate was positively associated with total needs satisfaction (i.e., the mediator), and total needs satisfaction was positively related to all eight life skills. The mastery climate was positively correlated with all eight life skills (i.e., the total effect was significant and positive). When including total needs satisfaction as the mediator, the direct effect of the mastery climate on all eight life skills was reduced, but remained statistically significant, suggesting partial mediation. These results indicated that total needs satisfaction partially mediated the relationships between the mastery climate and participants’ development of the eight life skills in PE. From Table 2, for all models, we can thus see that zero was not included within the lower and upper bound Cis for total needs satisfaction, suggesting mastery climate had an indirect effect on all eight life skills via total needs satisfaction.

For the models in Figure 3, the performance climate was positively associated with total needs frustration. Total needs frustration was negatively related to goal setting, social skills, and time management. However, there were no significant relationships between total needs frustration and teamwork, problem-solving and decision-making, emotional skills, leadership, and interpersonal communication. The total effect of the performance climate on all life skills besides interpersonal communication was not statistically significant. When including total needs frustration as the mediator, the direct effect of the performance climate on all eight life skills was not statistically significant. From the indirect effect in Table 3, for the models on teamwork, problem-solving and decision making, emotional skills, leadership, and interpersonal communication, we can see that zero was included within the lower and upper bound Cis for total needs frustration, whist zero was not included for the models on goal setting, social skills, and time management. Combined, these results suggested that total needs frustration did not mediate the relationship between performance climate and teamwork, problem-solving and decision making, emotional skills, leadership, and interpersonal communication. On the contrary, there was the masking effect (Wen and Ye, 2014) of the performance climate on goal setting, social skills, and time management via total needs frustration, but the effect sizes were very small.

## Discussion

The primary aim of the present study was to investigate how Chinese teachers develop students’ life skills in PE, using SDT and AGT (Standage et al., 2003; Jaakkola et al., 2015) as our theoretical framework. Specifically, we addressed the call for the following causal sequence to be investigated: perceived motivational climate affects participants’ BPNs, which in turn, influences the development of life skills (Brown and Fry, 2014; Cronin et al., 2022). Our study also responded to the call for investigations of both the ‘bright’ and ‘dark’ side variables of SDT on students’ development of life skills (Cronin et al., 2019, 2020). Furthermore, we attempted to discuss the cross-paths in the ‘bright’ and ‘dark’ sides, that no previous studies have investigated. In summary, this study extends prior research by supporting the idea that a mastery climate facilitates students’ satisfaction with their BPNs and consequently helps them to develop their life skills.

Our first hypothesis assessed the relationships between students’ perceptions of the teacher-initiated motivational climate and their BPNs. Our findings revealed that a mastery climate was positively related to total needs satisfaction and negatively related to total needs frustration. In comparison, a performance climate was negatively associated with total needs satisfaction and positively associated with total needs frustration. These results supported hypothesis 1. On the bright side, aligned with the antecedent findings (Garca-Gonzlez et al., 2019) and the SDT-based research (Leo et al., 2020; Cronin et al., 2022; Ji et al., 2022), these findings suggested mastery climate as well as autonomy and needs support as contextual variables could positively predict students’ BPNs satisfaction. As such, the more that students perceived the class climate to be positive, the more likely they were to feel that their BPNs were satisfied. Furthermore, the negative and significant cross-path from a mastery climate to total needs frustration supported past research indicating autonomy and needs support also negatively predicted BPNs frustration (Garca-Gonzlez et al., 2019; Leo et al., 2020; Cronin et al., 2022; Ji et al., 2022). According to Vansteenkiste and Ryan’s (2013) research revealing that needs-supportive circumstance could act a buffering part against poor well-being and malfunctioning, it may suggest that the positive contextual variables including mastery climate have effects on buffering BPNs frustration. On the dark side, the results also supported previous literature (Garca-Gonzlez et al., 2019) and the SDT-based research (Leo et al., 2020; Cronin et al., 2022; Ji et al., 2022). In line with need-tharwting or controlling behaviors, the more students perceived the class climate to be performance-oriented, the more likely they were to feel that their BPNs were frustrated. It may be because these teaching behaviors make pupils feel more pressure, inferior and being controlled. However, unlike previous findings (Garca-Gonzlez et al., 2019), this study and Ji et al.’s (2022) research showed a significant and negative relationship between performance climate, controlling teaching and Chinese students’ BPNs satisfaction. The possible explanation for these differing results is that in contrast to western education, Chinese education includes PE as a performance-focused, graded subject, which may lead to lower levels of students’ needs satisfaction and lower intrinsic motivation (Krijgsman et al., 2017), especially when the PE teacher overemphasizes grade-based success. Taken together, the results indicate that PE teachers should use instructional strategies that help to create a mastery climate and avoid creating a performance climate if they wish to satisfy students’ BPNs and buffer against needs frustration.

Our second hypothesis involved an evaluation of the relationship between students’ BPNs and their life skills development. Supporting hypothesis 2, total needs satisfaction was positively related, whereas total needs frustration was negatively associated, with students’ development of the eight life skills. The positive relationship between BPNs satisfaction and life skills development was consistent with previous cross-sectional (Cronin et al., 2019; Cheon et al., 2021; Ji et al., 2022) and longitudinal (Cronin et al., 2020) studies in PE, and also with Hodge et al.’s (2013) conceptual framework for life skills development. These findings indicated that satisfying students’ BPNs would positively predicts the development of life skills. Moreover, several SDT-based literature showed that BPNs satisfaction also predict other positive outcomes such as engagement (Leo et al., 2020), intention to physical activities and well-being (Behzadnia, 2020). Based on Vallerand’s (1997) SDT-based motivational sequence, it might be that greater BPNs satisfaction leads to higher levels of self-determined motivation, which, in turn, predicts positive outcomes and the development of life skills. Future studies should explore this proposition. Notably, in line with the studies by Cheon et al. (2021) and Ji et al. (2022), our results indicated that students’ BPNs frustration may inhibit their development of some life skills. However, this finding differed from Cronin et al.’s (2019) and Behzadnia’s (2020) study, which found no relationships between BPNs frustration and life skills development and well-being. The possible explanation for the difference in the results of needs frustration is due to the difference in the sampled groups of pupils in cultural and educational contexts or adopting differing research method. Future studies should look to further investigate why these different findings occur. Overall, our finding indicates that PE teachers should aim to create an environment where students’ three BPNs are satisfied to help develop their life skills.

For the third hypothesis, we assessed the relationships between the perceived motivational climate and students’ life skills development. In line with hypothesis 3, a perceived mastery-oriented climate was positively related to students’ eight life skills development, whereas a perceived performance climate was negatively correlated with it. The first finding was consistent with previous research (Di Battista et al., 2019; Rodrigues et al., 2020; Mossman et al., 2021), which showed that a mastery-oriented climate was positively associated with adaptive outcomes in PE, including life skills development. This finding also confirmed the implicit approach to life skills development and transfer (Bean et al., 2018). Specifically, a mastery climate was regarded as an implicit behavior that allowed participants to live experiences which could foster students’ life skills implicitly. However, previous research has suggested that a performance climate was associated with negative consequences (Serrano et al., 2016; Garca-Gonzlez et al., 2019). This was further confirmed in our study, as our second finding demonstrated that students’ learning of life skills may be hindered when PE teachers create a performance-oriented climate. Therefore, teachers’ inappropriate social interactions (e.g., creating a performance climate) may thwart the ability of young people to learn life skills (Kendellen and Camire, 2015; Newman et al., 2021). One explanation for this is that a performance climate causes participants to feel nervous, unappreciated, or focused on winning, and as a result, they are more likely to feel stressed and less likely to learn life skills (Neely and Holt, 2014). Nevertheless, it must also be noted that mastery climate was larger related to all eight life skills as compared to performance climate. This finding supported past studies in sport (Mossman et al., 2021) and PE (Garca-Gonzlez et al., 2019; Rodrigues et al., 2020) indicating mastery climate compared with performance had greater effects on positive outcomes (e.g., life skills development). Even so, teachers should still avoid performance-oriented behaviors in PE.

Our fourth hypothesis was to investigate the mediating role of BPNs in the relationships between the motivational climate and students’ life skills development. For the bright pathway, students’ total needs satisfaction partially mediated the relationships between a perceived mastery-oriented climate and students’ development of the eight life skills. Our findings confirmed hypothesis 4a and were consistent with previous studies showing that BPNs satisfaction mediated the relationships between a mastery climate and students’ adaptive outcomes in PE, such as their grades (Rodrigues et al., 2020), intention to engage in physical activity (Di Battista et al., 2019), moderate to vigorous physical activity (MVPA) (Chen et al., 2020), concentration (Mastagli et al., 2021) and motivation (Claver et al., 2020). Furthermore, our mediation model also supported the SDT-based research in PE (Cronin et al., 2019) and in sport (Cronin et al., 2022), which indicated that the more that students could perceive the class climate to be mastery-oriented as well as autonomy-supportive, the more likely they were to report higher levels of BPNs satisfaction, and, in turn, greater development of life skills. However, for the dark side, contrary to hypothesis 4b, we found that total needs frustration did not mediate the correlations between a performance-oriented climate and students’ development of all life skills besides goal setting, social skills, and time management, while the masking effect sizes of the performance climate on goal setting, social skills, and time management via needs frustration were very small. Several researchers indicated that students’ BPNs frustration had positive associations with negative outcomes in PE such as amotivation (Cheon et al., 2016), antisocial behavior (Cheon et al., 2018) and negative affect (Liu et al., 2017; Behzadnia et al., 2018). The possible explanation is that the experience of BPNs frustration mostly affects negative outcomes that may be obstructive factors of students’ developing life skills (Haerens et al., 2018; Cheon et al., 2021). To explain this phenomena, it needs to further explore whether existing other variable (e.g., motivation regulations) mediates the relationship between performance climate and life skills. Overall, our findings demonstrated that to satisfy students’ BPNs and develop their life skills, PE teachers should aim to create a mastery-oriented climate and avoid a performance climate.

### Practical implications

The finding from our study adds to a growing body of literature on adolescents’ life skills development, and assists in our understanding of the role of motivational climates on it. Specifically, mastery climate can be able to foster students’ satisfaction of BPNs, and, in turn, to help them to implicitly develop the following life skills: teamwork, goal setting, social skills, problem solving and decision making, emotional skills, leadership, time management, and interpersonal communication. This suggests that creating a mastery climate is an important skill for PE teachers.

Based on these findings, PE teachers ought to adopt some instructional strategies to build this climate. For instance, teachers provide students different choice of skill-level tasks, allow their mistakes and permit them to take part in decision-making about the content of lessons to meet students’ need for autonomy, and, in turn, to foster their goal setting, decision making and time management skills. Teachers set some activities of helping them master sports skills to promote them having more positive experiences, lead them to learn from errors and evaluate them through positive individualized feedback about their effort and progress, which could facilitate competence satisfaction, and, in turn, develop their problem solving and emotional skills. Teachers provide more opportunities for collaborative learning where each student performs a defined role to help others to improve abilities (e.g., through grouping), and concern and appreciate each student equally to meet their need for relatedness, and, in turn, to cultivate their teamwork, leadership and interpersonal communication skills. Meanwhile, it should also be noted that performance climate is negatively related to BPNs frustration and life skills development. Therefore, PE teachers ought to avoid such teaching behaviors of creating performance climate as well, such as overemphasizing on outperforming others, comparing them with others frequently, punishing their errors, focusing only on records, and concerning and endorsing only outstanding performer. Additionally, teachers ought to lead them how to apply these skills learning from PE to other circumstances (e.g., school, community, family).

### Limitations and future directions

Our study provided some novel and actionable findings but also had several limitations.

First, we used student self-reporting, yet we know that the truthfulness of responses and potential social desirability is always a limitation of this approach (Brenner and DeLamater, 2014). As such, future studies should use trained classroom observers (Cheon et al., 2018) or gain the perspectives of different observers (e.g., teachers, peers, parents) (Cronin et al., 2019), either to avoid this bias or to at least corroborate students’ ratings of the motivational climate, BPNs satisfaction, and life skills development in PE.

Second, the study involved a cross-sectional design, which could not examine causality. Given our promising cross-sectional findings, future life skills research based on AGT and SDT should verify our correlational findings via longitudinal and experimental research designs.

Third, our study focused only on teacher-created motivational climates. There might be the other-created climates that influence students’ life skills development in PE. For example, some studies suggested that a peer-created motivational climate may be a greater influence on adaptive PE outcomes (Rodrigues et al., 2020) included life skills development (Mossman et al., 2021). According to the different characteristic of teachers’ and peers’ motivational behaviors (Papaioannou, 1998; Ntoumanis and Vazou, 2005), future research ought to provide a comprehensive test of both motivational climates –created by teachers and peers – to see how they affect students’ life skills development.

Finally, Our study only considered two motivational climates as contextual factor variables, which could not be judged to have a greater impact on BPNs and life skills development than other factors. Based on previous SDT-based research (e.g., Cronin et al., 2019; Leo et al., 2020), future research could add autonomy-supportive and controlling teaching behaviors as contextual factors to test at the same time which is the better predictor (autonomy-supportive behavior or mastery climate) of needs satisfaction and life skills development, and which variables (controlling behavior or performance climate) better predict needs frustration.

## Conclusion

To the authors’ best knowledge, the current study is the first to utilize SDT and AGT to explore how students develop their life skills in PE. The present study supported and extended previous findings by finding the associations between students’ perceptions of teacher-initiated motivational climates and their life skills development. In particular, our findings showed that students’ BPNs satisfaction mediated the positive correlations between a mastery-oriented climate and their life skills development. However, our findings did not indicate that BPNs frustration mediated the correlations between a performance-oriented climate and their life skills besides goal setting, social skills, and time management. These findings highlight the importance of a mastery-oriented climate and psychological needs satisfaction for students to develop life skills in PE.

## Data availability statement

The raw data supporting the conclusions of this article will be made available by the authors, without undue reservation.

## Author contributions

SZ: conceptualization, software, formal analysis, investigation, resources, and writing—original draft preparation. SZ and XJ: methodology. SZ and LC: validation, writing—review and editing, visualization, and project administration. JX and XJ: data curation. XJ, LC, and JX: supervision. All authors contributed to the article and approved the submitted version.

## Conflict of interest

The authors declare that the research was conducted in the absence of any commercial or financial relationships that could be construed as a potential conflict of interest.

## Publisher’s note

All claims expressed in this article are solely those of the authors and do not necessarily represent those of their affiliated organizations, or those of the publisher, the editors and the reviewers. Any product that may be evaluated in this article, or claim that may be made by its manufacturer, is not guaranteed or endorsed by the publisher.

## References

[ref1] Ames C. (1992). “Achievement goals, motivational climate and motivational processes” in Motivation in sport and exercise. ed. Roberts G. C. (Champaign, Il: Human Kinetics), 161–176.

[ref2] Baard P. Deci E. L. Ryan R. M. (2004). Intrinsic need satisfaction: a motivational basis of performance and weil-being in two work settings. J. Appl. Soc. Psychol. 34, 2045–2068. doi: 10.1111/j.1559-1816.2004.tb02690.x

[ref3] Bean C. Forneris T. (2016). Examining the importance of intentionally structuring the youth sport context to facilitate positive youth development. J. Appl. Sport Psychol. 28, 410–425. doi: 10.1080/10413200.2016.1164764

[ref4] Bean C. Kramers S. Forneris T. Camire M. (2018). The implicit/explicit continuum of life skills development and transfer. Quest 70, 456–470. doi: 10.1080/00336297.2018.1451348

[ref5] Behzadnia B. (2020). The relations between students' causality orientations and teachers' interpersonal behaviors with students' basic need satisfaction and frustration, intention to physical activity, and well-being. Phys. Educ. Sport Peda. 26, 613–632. doi: 10.1080/17408989.2020.1849085

[ref6] Behzadnia B. Adachi P. J. C. Deci E. L. Mohammadzadeh H. (2018). Associations between students’ perceptions of physical education teachers’ interpersonal styles and students’ wellness, knowledge, performance, and intentions to persist at physical activity: a self-determination theory approach. Psychol. Sport Exerc. 39, 10–19. doi: 10.1016/j.psychsport.2018.07.003

[ref7] Brenner P. S. DeLamater J. D. (2014). Social desirability bias in self-reports of physical activity: is an exercise identity the culprit? Soc. Indic. Res. 117, 489–504. doi: 10.1007/s11205-013-0359-y

[ref8] Brown T. J. Fry M. D. (2014). Evaluating the pilot of strong girls: a life skills/physical activity program for third and fourth grade girls. J. Appl. Sport Psychol. 26, 52–65. doi: 10.1080/10413200.2013.778913

[ref9] Camire M. Rathwell S. Turgeon S. Kendellen K. (2019). Coach–athlete relationships, basic psychological needs satisfaction and thwarting, and the teaching of life skills in Canadian high school sport. Int. J. Sports Sci. Coa. 14, 591–606. doi: 10.1177/1747954119869542

[ref10] Chen R. Wang L. Wang B. Zhou Y. (2020). Motivational climate, need satisfaction, self-determined motivation, and physical activity of students in secondary school physical education in China. BMC Public Health 20:1687. doi: 10.1186/s12889-020-09750-x, PMID: 33172411 PMC7657358

[ref11] Cheon S. H. Reeve J. Ntoumanis N. (2018). A needs-supportive intervention to help pe teachers enhance students’ prosocial behavior and diminish antisocial behavior. Psychol. Sport Exerc. 35, 74–88. doi: 10.1016/j.psychsport.2017.11.010

[ref12] Cheon S. H. Reeve J. Song Y. G. (2016). A teacher-focused intervention to decrease pe students’ amotivation by increasing need satisfaction and decreasing need frustration. J. Sport Exercise Psy. 38, 217–235. doi: 10.1123/jsep.2015-0236 27385730

[ref13] Cheon S. Song Y. Yoo K. Joo W. Kim B. (2021). Effects of interpersonal types of life skills on the adolescent's in physical education: test mediated effect of psychological needs. Kor. Soc. Spor. Sci. 30, 319–334. doi: 10.35159/kjss.2021.2.30.1.319

[ref14] Chinkov A. E. Holt N. L. (2016). Implicit transfer of life skills through participation in brazilian jiu-jitsu. J. Appl. Sport Psychol. 28, 139–153. doi: 10.1080/10413200.2015.1086447

[ref15] Chu T. L. Zhang T. (2018). Motivational processes in sport education programs among high school students. Eur. Phys. Educ. Rev. 24, 372–394. doi: 10.1177/1356336x17751231

[ref16] Claver F. Martinez-Aranda L. M. Conejero M. Gil-Arias A. (2020). Motivation, discipline, and academic performance in physical education: a holistic approach from achievement goal and self-determination theories. Front. Psychol. 11:1808. doi: 10.3389/fpsyg.2020.01808, PMID: 32903702 PMC7438928

[ref17] Cohen J. (1988). Statistical power analysis for the behavioural sciences. Hillsdale, Nj: Erlbaum.

[ref18] Cronin L. Allen J. (2017). Development and initial validation of the life skills scale for sport. Psychol. Sport Exerc. 28, 105–119. doi: 10.1016/j.psychsport.2016.11.001

[ref19] Cronin L. Allen J. Mulvenna C. Russell P. (2018). An investigation of the relationships between the teaching climate, students’ perceived life skills development and well-being within physical education. Phys. Educ. Sport Peda. 23, 181–196. doi: 10.1080/17408989.2017.1371684

[ref20] Cronin L. Ellison P. Allen J. Huntley E. Johnson L. Kosteli M. C.. (2022). A self-determination theory based investigation of life skills development in youth sport. J. Sport Sci. 40, 886–898. doi: 10.1080/02640414.2022.2028507 35060436

[ref21] Cronin L. Marchant D. Allen J. Mulvenna C. Cullen D. Williams G.. (2019). Students’ perceptions of autonomy-supportive versus controlling teaching and basic need satisfaction versus frustration in relation to life skills development in pe. Psychol. Sport Exerc. 44, 79–89. doi: 10.1016/j.psychsport.2019.05.003

[ref22] Cronin L. Marchant D. R. Johnson L. A. Huntley E. Kosteli M. Varga J.. (2020). Life skills development in physical education: a self-determination theory-based investigation across the school term. Psychol. Sport Exerc. 49:101711. doi: 10.1016/j.psychsport.2020.101711

[ref23] De Meyer J. Soenens B. Vansteenkiste M. Aelterman N. Van Petegem S. Haerens L. (2016). Do students with different motives for physical education respond differently to autonomy-supportive and controlling teaching? Psychol. Sport Exerc. 22, 72–82. doi: 10.1016/j.psychsport.2015.06.001

[ref24] De Ridder D. T. D. Lensvelt-mulders G. Finkenauer C. Stok F. M. Baumeister R. F. (2018). Taking stock of self-control: a meta-analysis of how trait self-control relates to a wide range of behaviors. Personal. Soc. Psychol. Rev. 16, 76–99. doi: 10.1177/1088868311418749, PMID: 21878607

[ref25] Deci E. L. Ryan R. M. (2008). Facilitating optimal motivation and psychological well-being across life’s domains. Can. Psychol. 49, 14–23. doi: 10.1037/0708-5591.49.1.14

[ref26] Deci E. L. Ryan R. M. (2014). “Self-determination theory” in Sage publications ltd Ebooks, 416–437. doi: 10.4135/9781446249215.n21

[ref27] Di Battista R. Robazza C. Ruiz M. C. Bertollo M. Vitali F. Bortoli L. (2019). Student intention to engage in leisure-time physical activity: the interplay of task-involving climate, competence need satisfaction and psychobiosocial states in physical education. Eur. Phys. Educ. Rev. 25, 761–777. doi: 10.1177/1356336x18770665

[ref28] Duda J. L. (2001). “Goal perspectives and their implications for health related outcomes in the physical domain” in Advances in motivation theories in the sport domain. eds. Cury F. Sarrazin P. Famose F. P. (Paris: Presses Universitaires de France)

[ref30] Garca-Gonzlez L. Sevil-Serrano J. Abs A. Aelterman N. Haerens L. (2019). The role of task and ego-oriented climate in explaining students’ bright and dark motivational experiences in physical education. Phys. Educ. Sport Peda. 24, 344–358. doi: 10.1080/17408989.2019.1592145

[ref31] Girard S. Lemoyne J. Blais D. St-Amand J. (2021). An analysis of mechanisms underlying social goals in physical education: a comparison between ordinary and special classes. Phys. Educ. Sport Peda. 27, 320–337. doi: 10.1080/17408989.2021.1879767

[ref32] Gjesdal S. Stenling A. Solstad B. E. Ommundsen Y. (2019). A study of coach-team perceptual distance concerning the coach-created motivational climate in youth sport. Scand. J. Med. Sci. Spor. 29, 132–143. doi: 10.1111/sms.13306, PMID: 30230049

[ref33] Goudas M. Dermitzaki I. Leondari A. Danish S. J. (2006). The effectiveness of teaching a life skills program in a physical education context. Eur. J. Psychol. Educ. 21, 429–438. doi: 10.1007/bf03173512

[ref34] Goudas M. Giannoudis G. (2008). A team-sports-based life-skills program in a physical education context. Learn. Instr. 18, 528–536. doi: 10.1016/j.learninstruc.2007.11.002

[ref35] Gould D. Carson S. (2008). Life skills development through sport: current status and future directions. Int. Rev. Sport Exer. P. 1, 58–78. doi: 10.1080/17509840701834573

[ref36] Gould D. Flett R. M. Lauer L. (2012). The relationship between psychosocial developmental and the sports climate experienced by underserved youth. Psychol. Sport Exerc. 13, 80–87. doi: 10.1016/j.psychsport.2011.07.005

[ref37] Haerens L. Aelterman N. Vansteenkiste M. Soenens B. Van Petegem S. (2015). Do perceived autonomy-supportive and controlling teaching relate to physical education students’ motivational experiences through unique pathways? Distinguishing between the bright and dark side of motivation. Psychol. Sport Exerc. 16, 26–36. doi: 10.1016/j.psychsport.2014.08.013

[ref38] Haerens L. Vansteenkiste M. De Meester A. Delrue J. Tallir I. Vande Broek G.. (2018). Different combinations of perceived autonomy support and control: identifying the most optimal motivating style. Phys. Educ. Sport Peda. 23, 16–36. doi: 10.1080/17408989.2017.1346070

[ref39] Harwood C. G. Keegan R. Smith J. D. Raine A. S. (2015). A systematic review of the intrapersonal correlates of motivational climate perceptions in sport and physical activity. Psychol. Sport Exerc. 18, 9–25. doi: 10.1016/j.psychsport.2014.11.005

[ref40] Hayes A.F. (2013). Introduction to mediation, moderation, and conditional process analysis: A regression-based approach. New York, NY: Guilford Publications.

[ref41] Hodge K. Danish S. J. Forneris T. Miles A. (2016). “Life skills and basic psychological needs” in Routledge Ebooks, 45–56. doi: 10.4324/9781315709499-5

[ref42] Hodge K. Danish S. Martin J. (2013). Developing a conceptual framework for life skills interventions. Couns. Psychol. 41, 1125–1152. doi: 10.1177/0011000012462073

[ref43] Hodge K. Gucciardi D. F. (2015). Antisocial and prosocial behavior in sport: the role of motivational climate, basic psychological needs, and moral disengagement. J. Sport Exercise Psy. 37, 257–273. doi: 10.1123/jsep.2014-0225, PMID: 26265339

[ref44] Holt N. L. Neely K. C. Slater L. Camiré M. Côté J. Fraser-Thomas J.. (2017). A grounded theory of positive youth development through sport based on results from a qualitative meta-study. Int. Rev. Sport Exer. P. 10, 1–49. doi: 10.1080/1750984x.2016.1180704, PMID: 27695511 PMC5020349

[ref45] Holt N. L. Tink L. N. Mandigo J. L. Fox K. R. (2008). Do youth learn life skills through their involvement in high school sport? A case study. Can. J. Educ. 31, 281–304. Available at: https://journals.sfu.ca/cje/index.php/cje-rce/article/view/3003.

[ref46] IBM Corporation (2017). SPSS statistics for windows. Armonk, NY: IBM Corporation Version 25.0.

[ref47] Jaakkola T. Wang C. J. Soini M. Liukkonen J. (2015). Students’ perceptions of motivational climate and enjoyment in finnish physical education: a latent profile analysis. J. Sport Sci. Med. 14, 477–483. Available at: https://www.jssm.org/volume14/iss3/cap/jssm-14-477.pdf. PMC4541109 26336332

[ref48] Jacobs J. M. Wright P. M. Richards K. A. R. (2022). Students’ perceptions of learning life skills through the teaching personal and social responsibility model: an exploratory study. Front. Sports Act. Living. 4:898738. doi: 10.3389/fspor.2022.898738, PMID: 35711854 PMC9197241

[ref49] Ji X. Zheng S. Cheng C. Cheng L. Cronin L. (2022). Development and psychometric evaluation of the chinese version of the life skills scale for physical education. Int. J. Env. Res. Pub. He. 19:5324. doi: 10.3390/ijerph19095324, PMID: 35564715 PMC9104646

[ref50] Kendellen K. Camire M. (2015). Examining former athletes’ developmental experiences in high school sport. SAGE Open 5:215824401561437. doi: 10.1177/2158244015614379

[ref51] Krijgsman C. Vansteenkiste M. Van Tartwijk J. Maes J. Borghouts L. Cardon G.. (2017). Performance grading and motivational functioning and fear in physical education: a self-determination theory perspective. Learn. Individ. Differ. 55, 202–211. doi: 10.1016/j.lindif.2017.03.017

[ref52] Larson R. W. Hansen D. M. Moneta G. (2006). Differing profiles of developmental experiences across types of organised youth activities. Dev. Psychol. 42, 849–863. doi: 10.1037/0012-1649.42.5.849, PMID: 16953691

[ref53] Leo F. M. Mouratidis A. Pulido J. J. Lopez-Gajardo M. A. Sanchez-Oliva D. (2020). Perceived teachers' behavior and students' engagement in physical education: the mediating role of basic psychological needs and self-determined motivation. Phys. Educ. Sport Peda. 27, 59–76. doi: 10.1080/17408989.2020.1850667

[ref54] Liu J. Bartholomew K. J. Chung P. (2017). Perceptions of teachers’ interpersonal styles and well-being and ill-being in secondary school physical education students: the role of need satisfaction and need frustration. Sch. Ment. Health. 9, 360–371. doi: 10.1007/s12310-017-9223-6

[ref55] Liu J. Chung P. (2014). Development and initial validation of the psychological needs satisfaction scale in physical education. Meas. Phys. Educ. Exerc. 18, 101–122. doi: 10.1080/1091367x.2013.872106

[ref56] Liu J. Chung P. (2015). Development and initial validation of the chinese version of psychological needs thwarting scale in physical education. J. Teach. Phys. Educ. 34, 402–423. doi: 10.1123/jtpe.2014-0053

[ref57] Malete L. McCole D. Tshube T. Mphela T. Maro C. Adamba C.. (2022). Effects of a sport-based positive youth development program on youth life skills and entrepreneurial mindsets. PLoS One 17:e0261809. doi: 10.1371/journal.pone.0261809, PMID: 35120126 PMC8815907

[ref58] Maro C. N. Roberts G. (2012). Combating hiv/aids in sub-saharan africa: effect of introducing a mastery motivational climate in a community-based programme. Appl. Psychol-Int. Rev. 61, 699–722. doi: 10.1111/j.1464-0597.2011.00482.x

[ref59] Marsh H. W. (1992). Extracurricular activities: beneficial extension of the traditional curriculum or subversion of academic goals? J. Educ. Psychol. 84, 553–562. doi: 10.1037/0022-0663.84.4.553

[ref60] Mastagli M. Van Hoye A. Hainaut J. Bolmont B. (2021). The role of an empowering motivational climate on pupils’ concentration and distraction in physical education. J. Teach. Phys. Educ. 41, 311–321. doi: 10.1123/jtpe.2020-0252

[ref61] Moreau E. Mageau G. A. (2012). The importance of perceived autonomy support for the psychological health and work satisfaction of health professionals: not only supervisors count, colleagues too! Motiv. Emotion. 36, 268–286. doi: 10.1007/s11031-011-9250-9

[ref62] Mossman G. Robertson C. F. Williamson B. D. Cronin L. (2021). Coaches, parents, or peers: who has the greatest influence on sports participants’ life skills development? J. Sport Sci. 39, 2475–2484. doi: 10.1080/02640414.2021.1939980 34130606

[ref63] Neely K. C. Holt N. L. (2014). Parents’ perspectives on the benefits of sport participation for young children. Sport Psychol. 28, 255–268. doi: 10.1123/tsp.2013-0094

[ref64] Newman T. J. Anderson-Butcher D. Amorose A. J. (2020). Examining the influence of sport program staff and parent/caregiver support on youth outcomes. Appl. Dev. Sci. 24, 263–278. doi: 10.1080/10888691.2018.1467762

[ref65] Newman T. Black S. Santos F. Jefka B. Brennan N. (2021). Coaching the development and transfer of life skills: a scoping review of facilitative coaching practices in youth sports. Int. Rev. Sport Exer. 1–38. doi: 10.1080/1750984x.2021.1910977

[ref66] Ntoumanis N. Vazou S. (2005). Peer motivational climate in youth sport: measurement development and validation. J. Sport Exercise. Psy. 27, 432–455. doi: 10.1123/jsep.27.4.432

[ref67] Opstoel K. Chapelle L. Prins F. J. De Meester A. Haerens L. Van Tartwijk J.. (2019). Personal and social development in physical education and sports: a review study. Eur. Phys. Educ. Rev. 26, 797–813. doi: 10.1177/1356336x19882054

[ref68] Papaioannou A. (1998). Students' perceptions of the physical education class environment for boys and girls and the perceived motivational climate. Res. Q. Exercise Sport. 69, 267–275. doi: 10.1080/02701367.1998.10607693, PMID: 9777663

[ref69] Pesce C. Marchetti R. Forte R. Crova C. Scatigna M. Goudas M.. (2016). Youth life skills training: exploring outcomes and mediating mechanisms of a group-randomized trial in physical education. Sport Exerc. Perform. 5, 232–246. doi: 10.1037/spy0000060

[ref70] Petitpas A. J. Cornelius A. E. Van Raalte J. L. Jones T. (2005). A framework for planning youth sport programs that foster psychosocial development. Sport Psychol. 19, 63–80. doi: 10.1123/tsp.19.1.63

[ref71] Pierce S. Gould D. Camiré M. (2017). Definition and model of life skills transfer. Int. Rev. Sport Exer. P. 10, 186–211. doi: 10.1080/1750984x.2016.1199727

[ref72] Preacher K. J. Hayes A. F. (2004). SPSS and SAS procedures for estimating indirect effects in simple mediation models. Behav. Res. Methods 36, 717–731. doi: 10.3758/BF03206553, PMID: 15641418

[ref73] Reinboth M. Duda J. L. (2006). Perceived motivational climate, need satisfaction and indices of well-being in team sports: a longitudinal perspective. Psychol. Sport Exerc. 7, 269–286. doi: 10.1016/j.psychsport.2005.06.002

[ref74] Roberts G. C. Treasure D. C. Conroy D. E. (2012). “Understanding the dynamics of motivation in sport and physical activity: an achievement goal interpretation” in Handbook of sport psychology, 1–30. doi: 10.1002/9781118270011.ch1

[ref75] Rodrigues F. Monteiro D. Teixeira D. S. Cid L. (2020). The relationship between teachers and peers’ motivational climates, needs satisfaction, and physical education grades: an AGT and SDT approach. Int. J. Env. Res. Pub. He. 17:6145. doi: 10.3390/ijerph17176145, PMID: 32847056 PMC7504719

[ref76] Ryan R. M. Deci E. L. (2008). A self-determination theory approach to psychotherapy: the motivational basis for effective change. Can. Psychol. 49, 186–193. doi: 10.1037/a0012753

[ref77] Ryan R.M. Deci E.L. (2017). Self-determination theory: Basic psychological needs in motivation, development, and wellness. New York: Guilford Press Ebooks.

[ref78] Sackett S. C. Gano-Overway L. (2017). Coaching life skills development: best practices and high school tennis coach exemplar. Int. Sport Coach J. 4, 206–219. doi: 10.1123/iscj.2016-0080

[ref79] Santos F. Bean C. Azevedo N. Cardoso A. Pereira P. Cruz H. (2020). Moving from an implicit to an explicit approach of life skills development and transfer: the case of surfing in schools. SAGE Open 10:215824402093331. doi: 10.1177/2158244020933316

[ref80] Santos F. D. S. Camiré M. Campos P. H. D. (2016). Youth sport coaches’ role in facilitating positive youth development in portuguese field hockey. Int. J. Sport Exerc. Ps. 16, 221–234. doi: 10.1080/1612197x.2016.1187655

[ref81] Schweizer G. Furley P. (2016). Reproducible research in sport and exercise psychology: the role of sample sizes. Psychol. Sport Exerc. 23, 114–122. doi: 10.1016/j.psychsport.2015.11.005 42480806

[ref82] Serrano J. Solana A. A. Catalan A. A. González L. A. (2016). Motivational climate of teaching physical education: could it affect student grades? Retos: Nuevas Tendencias En Educación Física. Deportes y Recreación. 31, 98–102. doi: 10.47197/retos.v0i31.46514

[ref83] Sheldon K. M. Niemiec C. P. (2006). It’s not just the amount that counts: balanced need satisfaction also affects well-being. J. Pers. Soc. Psychol. 91, 331–341. doi: 10.1037/0022-3514.91.2.331 16881768

[ref84] Sheridan D. Coffee P. Lavallee D. (2014). A systematic review of social support in youth sport. Int. Rev. Sport Exer. P. 7, 198–228. doi: 10.1080/1750984x.2014.931999

[ref85] Sim M. Kim S. Y. Suh Y. (2021). Sample size requirements for simple and complex mediation models. Educ. Psychol. Meas. 82, 76–106. doi: 10.1177/00131644211003261, PMID: 34992307 PMC8725051

[ref86] Singla D. R. Waqas A. Hamdani S. U. Suleman N. Zafar S. W. Zill-E-Huma. (2020). Implementation and effectiveness of adolescent life skills programs in low- and middle-income countries: a critical review and meta-analysis. Behav. Res. Ther. 130:103402. doi: 10.1016/j.brat.2019.04.010 31146889

[ref87] Smith R. E. Cumming S. P. Smoll F. L. (2008). Development and validation of the motivational climate scale for youth sports. J. Appl. Sport Psychol. 20, 116–136. doi: 10.1080/10413200701790558

[ref88] Smith N. Quested E. Appleton P. R. Duda J. L. (2016). A review of observational instruments to assess the motivational environment in sport and physical education settings. Int. Rev. Sport Exer. P. 9, 134–159. doi: 10.1080/1750984x.2015.1132334

[ref89] Standage M. Duda J. L. Ntoumanis N. (2003). A model of contextual motivation in physical education: using constructs from self-determination and achievement goal theories to predict physical activity intentions. J. Educ. Psychol. 95, 97–110. doi: 10.1037/0022-0663.95.1.97

[ref90] Tabachnick B.G. Fidell L.S. (2013). Using multivariate statistics (6th ed.) Boston, MA: Pearson Education Inc.

[ref91] The Ministry of Education of the People’s Republic of China (2018) Curriculum standard for physical education and health in senior high schools (2017th) Beijing: People’s Education Press:5.

[ref92] Treasure D. C. Roberts G. C. (2001). Students’ perceptions of the motivational climate, achievement beliefs, and satisfaction in physical education. Res. Q. Exerc. Sport. 72, 165–175. doi: 10.1080/02701367.2001.10608946, PMID: 11393879

[ref93] Turnnidge J. Cote J. Hancock D. J. (2014). Positive youth development from sport to life: explicit or implicit transfer? Quest 66, 203–217. doi: 10.1080/00336297.2013.867275

[ref94] Vallerand R.J. (1997). Toward a hierarchical model of intrinsic and extrinsic motivation. New York: Elsevier Ebooks. 29.

[ref95] Vansteenkiste M. Ryan R. M. (2013). On psychological growth and vulnerability: basic psychological need satisfaction and need frustration as a unifying principle. J. Psychother. Integr. 23, 263–280. doi: 10.1037/a0032359

[ref96] Weeldenburg G. Borghouts L. Slingerland M. Vos S. (2020). Similar but different: profiling secondary school students based on their perceived motivational climate and psychological need-based experiences in physical education. PLoS One 15:e0228859. doi: 10.1371/journal.pone.0228859, PMID: 32040543 PMC7010287

[ref97] Wen Z. L. Ye B. J. (2014). Mediating effect analysis: methodology and model development. Adv. Psychol. Sci. 22, 731–745. doi: 10.3724/SP.J.1042.2014.00731

[ref98] Williams G. C. Grow V. M. Freedman Z. B. Ryan R. M. Deci E. L. (1996). Motivational predictors of weight loss and weight-loss maintenance. J. Pers. Soc. Psychol. 70, 115–126. doi: 10.1037/0022-3514.70.1.115 8558405

[ref99] Williams C. K. Neil R. Cropley B. Woodman T. Roberts R. (2020). A systematic review of sport-based life skills programs for young people: the quality of design and evaluation methods. J. Appl. Sport Psychol. 34, 409–435. doi: 10.1080/10413200.2020.1792583

[ref100] Wu X. Gai X. Yu T. Yu H. Zhang Y. (2021). Perceived motivational climate and stages of exercise behavior change: mediating roles of motivation within and beyond physical education class. Front. Psychol. 12:737461. doi: 10.3389/fpsyg.2021.737461, PMID: 34759869 PMC8573023

[ref001] Zins J. E. Weissberg R. P. Wang M. C. Walberg H. J. (2004). Building academic success on social and emotional learning: What does the research say?. New York: Teachers College Press., PMID:

