# Supplementary material for: Perceptions of the motivational climate, basic psychological needs, and life skills development in Chinese physical education students
Source: Front Psychol. 2023 Aug 15;14:1232849. doi: 10.3389/fpsyg.2023.1232849 (PMC10462985; doi:10.3389/fpsyg.2023.1232849)
Supplement: Supplementary file 1 [file Data_Sheet_1.pdf]

Dear students,

Life skills are key skills for teens to live healthy lives and get ahead. Our research group is conducting a study on the relationship between perception of the motivational climate in physical education and the development of life skills. Thank you very much for participating in this survey. This survey adopts anonymous method, in line with the principle of voluntary, there is no right or wrong choice, the results are only used as research, will not bring any influence to you, you do not have any concerns, realistic answer, do not omit to select any items. Thank you.

Part 1: Your basic situation

1. Your gender: Male ( ) female ( );
2. Your age: _______;
3. Which grade are you currently in:
   Junior one ( ); Junior two ( ); Junior three ( );
   Senior One ( ); Senior Two (); Senior Three ( ).
4. How many PE classes do you take per week? ________
   How minutes is the time of each PE class? ________
5. What sports did you learn in PE class this semester? ____________
6. Do you participate in any physical activities other than physical education (if you select "Yes", please continue to answer questions 7 and 8);
   Yes ( ); No ( )
7. If yes, which sports do you participate in other than physical education Classes: ______
8. The amount of time you normally spend participating in physical activities other than PE this semester is approximately minutes per week: _____

Part 2

Read each statement carefully and tick " √ " in the box that best represents your true psychological feelings in the physical education class so far this semester or so far. Please be sure to take the time to think carefully and answer truthfully through your true psychological feelings without fear. The answer time should be about 10-15 minutes. Your answers will help us carry out our scientific research. Thank you for your participation and support.

Motivational Climate Scale

1. The teacher made players feel good when they improved a skill.

1	2	3	4	5
Not at all True	A Little True	Somewhat True	A Lot True	Very True

2. The teacher encouraged us to learn new skills.

1	2	3	4	5
Not at all True	A Little True	Somewhat True	A Lot True	Very True

3. The teacher told players to help each other get better.

1	2	3	4	5
Not at all True	A Little True	Somewhat True	A Lot True	Very True

4. The teacher told us that trying our best was the most important thing.

1	2	3	4	5
Not at all True	A Little True	Somewhat True	A Lot True	Very True

5. Teacher said that teammates should help each other improve their skills.

1	2	3	4	5
Not at all True	A Little True	Somewhat True	A Lot True	Very True

6. The teacher said that all of us were important to the team's success.

1	2	3	4	5
Not at all True	A Little True	Somewhat True	A Lot True	Very True

7. Winning games was the most important thing for the teacher.

1	2	3	4	5
Not at all True	A Little True	Somewhat True	A Lot True	Very True

8. The teacher spent less time with the players who weren't as good.

1	2	3	4	5
Not at all True	A Little True	Somewhat True	A Lot True	Very True

9. The teacher told us which players on the team were the best.

1	2	3	4	5
Not at all True	A Little True	Somewhat True	A Lot True	Very True

10. The teacher paid most attention to the best players.

1	2	3	4	5
Not at all True	A Little True	Somewhat True	A Lot True	Very True

11. Teacher told us to try to be better than our teammates.

1	2	3	4	5
Not at all True	A Little True	Somewhat True	A Lot True	Very True

12. Players were taken out of games if they made a mistake.

1	2	3	4	5
Not at all True	A Little True	Somewhat True	A Lot True	Very True

Psychological Needs Scale

1. I have the opportunities to express my views and thoughts in my physical education classes.

1	2	3	4	5	6	7
Strongly Disagree	Disagree	Disagree Somewhat	Neutral	Agree Somewhat	Agree Strongly	Disagree

2. I am doing the activities that I really like in my physical education classes.

1	2	3	4	5	6	7
Strongly Disagree	Disagree	Disagree Somewhat	Neutral	Agree Somewhat	Agree Strongly	Disagree

3. I participate in physical education classes based on my personal preference.

1	2	3	4	5	6	7
Strongly Disagree	Disagree	Disagree Somewhat	Neutral	Agree Somewhat	Agree Strongly	Disagree

4. I am free to do physical activities the way I like in my physical education classes.

1	2	3	4	5	6	7
Strongly Disagree	Disagree	Disagree Somewhat	Neutral	Agree Somewhat	Agree Strongly	Disagree

5. I get opportunities to feel that I am good at sport in my physical education classes.

1	2	3	4	5	6	7
Strongly Disagree	Disagree	Disagree Somewhat	Neutral	Agree Somewhat	Agree Strongly	Disagree

6. I have the ability to perform well in my physical education classes.

1	2	3	4	5	6	7
Strongly Disagree	Disagree	Disagree Somewhat	Neutral	Agree Somewhat	Agree Strongly	Disagree

7. People in my physical education classes consider me good at sports.

1	2	3	4	5	6	7
Strongly Disagree	Disagree	Disagree Somewhat	Neutral	Agree Somewhat	Agree Strongly	Disagree

8. I feel comfortable when being with the people in my physical education classes.

1	2	3	4	5	6	7
Strongly Disagree	Disagree	Disagree Somewhat	Neutral	Agree Somewhat	Agree Strongly	Disagree

9. I interact friendly with the people in my physical education classes.

1	2	3	4	5	6	7
Strongly Disagree	Disagree	Disagree Somewhat	Neutral	Agree Somewhat	Agree Strongly	Disagree

10. I get along well with the people in my physical education classes.

1	2	3	4	5	6	7
Strongly Disagree	Disagree	Disagree Somewhat	Neutral	Agree Somewhat	Agree Strongly	Disagree

11. I feel pushed to behave in certain ways in my physical education classes.

1	2	3	4	5	6	7
Strongly Disagree	Disagree	Disagree Somewhat	Neutral	Agree Somewhat	Agree Strongly	Disagree

12. I often feel like I have to follow other people's commands in my physical education classes.

1	2	3	4	5	6	7

Strongly Disagree Disagree Disagree Somewhat Neutral Agree Somewhat Agree Strongly Disagree

13. I often feel that I am being forced to do things that I don't want to do in my physical education classes.

1 2 3 4 5 6 7

Strongly Disagree Disagree Disagree Somewhat Neutral Agree Somewhat Agree Strongly Disagree

14. I often feel like I am inadequate in my physical education classes.

1 2 3 4 5 6 7

Strongly Disagree Disagree Disagree Somewhat Neutral Agree Somewhat Agree Strongly Disagree

15. There are situations where I am made to feel inadequate in my physical education classes.

1 2 3 4 5 6 7

Strongly Disagree Disagree Disagree Somewhat Neutral Agree Somewhat Agree Strongly Disagree

16. I often doubt whether I am able to execute the tasks in my physical education properly.

1 2 3 4 5 6 7

Strongly Disagree Disagree Disagree Somewhat Neutral Agree Somewhat Agree Strongly Disagree

17. I feel others in my physical education classes can be dismissive of me.

1 2 3 4 5 6 7

Strongly Disagree Disagree Disagree Somewhat Neutral Agree Somewhat Agree Strongly Disagree

18. I feel some people in my physical education classes do not like me much.

1 2 3 4 5 6 7

Strongly Disagree Disagree Disagree Somewhat Neutral Agree Somewhat Agree Strongly Disagree

19. I feel I am alone in my physical education classes.

1 2 3 4 5 6 7

Strongly Disagree Disagree Disagree Somewhat Neutral Agree Somewhat Agree Strongly Disagree

Life Skills Development Scale

PE classes have taught me to...

1. Accept suggestions for improvement from others

1 2 3 4 5

Not at all A little Some A lot Very much

2. Help build team/group spirit

1 2 3 4 5

Not at all A little Some A lot Very much

3. Work well within a team/group

1 2 3 4 5
Not at all A little Some A lot Very much

4. Suggest to team/group members how they can improve their performance
1 2 3 4 5
Not at all A little Some A lot Very much

5. Help another team/group member perform a task
1 2 3 4 5
Not at all A little Some A lot Very much

6. Change the way I perform for the benefit of the team/group
1 2 3 4 5
Not at all A little Some A lot Very much

7. Work with others for the good of the team/group
1 2 3 4 5
Not at all A little Some A lot Very much

8. Set goals so that I can stay focused on improving
1 2 3 4 5
Not at all A little Some A lot Very much

9. Set challenging goals
1 2 3 4 5
Not at all A little Some A lot Very much

10. Check progress towards my goals
1 2 3 4 5
Not at all A little Some A lot Very much

11. Set short-term goals in order to achieve long-term goals
1 2 3 4 5
Not at all A little Some A lot Very much

12. Remain committed to my goals
1 2 3 4 5
Not at all A little Some A lot Very much

13. Set goals for practice
1 2 3 4 5
Not at all A little Some A lot Very much

14. Set specific goals

1 2 3 4 5
Not at all A little Some A lot Very much

15. Interact in various social settings
1 2 3 4 5
Not at all A little Some A lot Very much

16. Maintain close friendships
1 2 3 4 5
Not at all A little Some A lot Very much

17. Start a conversation
1 2 3 4 5
Not at all A little Some A lot Very much

18. Get involved in group activities
1 2 3 4 5
Not at all A little Some A lot Very much

19. Help others without them asking for help
1 2 3 4 5
Not at all A little Some A lot Very much

20. Think carefully about a problem
1 2 3 4 5
Not at all A little Some A lot Very much

21. Create as many possible solutions to a problem as possible
1 2 3 4 5
Not at all A little Some A lot Very much

22. Compare each possible solution in order to find the best one
1 2 3 4 5
Not at all A little Some A lot Very much

23. Evaluate a solution to a problem
1 2 3 4 5
Not at all A little Some A lot Very much

24. Know how to deal with my emotions
1 2 3 4 5
Not at all A little Some A lot Very much

25. Use my emotions to stay focused

1 2 3 4 5
Not at all A little Some A lot Very much

26. Understand that I behave differently when emotional
1 2 3 4 5
Not at all A little Some A lot Very much

27. Notice how I feel
1 2 3 4 5
Not at all A little Some A lot Very much

28. Set high standards for the team/group
1 2 3 4 5
Not at all A little Some A lot Very much

29. Know how to motivate others
1 2 3 4 5
Not at all A little Some A lot Very much

30. Help others solve their performance problems
1 2 3 4 5
Not at all A little Some A lot Very much

31. Be a good role model for others
1 2 3 4 5
Not at all A little Some A lot Very much

32. Organise team/group members to work together
1 2 3 4 5
Not at all A little Some A lot Very much

33. Recognise other peoples' achievements
1 2 3 4 5
Not at all A little Some A lot Very much

34. Know how to positively influence a group of individuals
1 2 3 4 5
Not at all A little Some A lot Very much

35. Consider the individual opinions of each team/group member
1 2 3 4 5
Not at all A little Some A lot Very much

36. Manage my time well

1 2 3 4 5
Not at all A little Some A lot Very much

37. Assess how much time I spend on various activities
1 2 3 4 5
Not at all A little Some A lot Very much

38. Control how I use my time
1 2 3 4 5
Not at all A little Some A lot Very much

39. Set goals so that I use my time effectively
1 2 3 4 5
Not at all A little Some A lot Very much

40. Speak clearly to others
1 2 3 4 5
Not at all A little Some A lot Very much

41. Pay attention to what someone is saying
1 2 3 4 5
Not at all A little Some A lot Very much

42. Pay attention to peoples' body language
1 2 3 4 5
Not at all A little Some A lot Very much

43. Communicate well with others
1 2 3 4 5
Not at all A little Some A lot Very much

This is the end of the questionnaire. Thank you again for your support and participation
